# Correlation Between Electronic Patient-Reported Outcomes and Biological Markers of Key Parameters in Acute Radiation Cystitis Among Patients With Prostate Cancer (RABBIO): Prospective Observational Study

**DOI:** 10.2196/48225

**Published:** 2024-12-12

**Authors:** Carole Helissey, Sophie Cavallero, Nathalie Guitard, Hélène Thery, Charles Parnot, Antoine Schernberg, Imen Aissa, Florent Raffin, Christine Le Coz, Stanislas Mondot, Christos Christopoulos, Karim Malek, Emmanuelle Malaurie, Pierre Blanchard, Cyrus Chargari, Sabine Francois

**Affiliations:** 1 Clinical Unit Research, Military Hospital Begin, 69 avenue de Paris, Saint-Mandé, 94240, France, 33 679526487; 2 Department of Radiation Biological Effects, French Armed Forces Biomedical Research Institute, IRBA, Brétigny-sur-Orge, France; 3 Cureety, Dinan, France; 4 MEDIAXE, CLAMART, France; 5 Department of Radiation Biological Effects, French Armed Forces Biomedical Research Institute, Brétigny-sur-Orge, France; 6 Paris-Saclay University, INRAE, AgroParisTech, Micalis Institute, Jouy-en-Josas, France; 7 Department of Radiotherapy, Le Raincy Montfermeil General Hospital, Montfermeil, France; 8 Department of Radiotherapy, Centre Hospitalier Intercommunal de Créteil, Créteil, France; 9 Department of Radiation Oncology, Gustave Roussy Cancer Campus, Université Paris-Saclay, Oncostat U1018 INSERM, Gustave-Roussy, Villejuif, France; 10 Department of Radiation Oncology, Pitié Salpêtrière University Hospital, Paris, France

**Keywords:** prostate cancer, acute radiation cystitis, e-PRO, quality of life, biomarkers, electronic patient-reported outcome

## Abstract

**Background:**

Despite advances in radiation techniques, radiation cystitis (RC) remains a significant cause of morbidity from pelvic radiotherapy, which may affect patients’ quality of life (QoL). The pathophysiology of RC is not well understood, which limits the development of effective treatments.

**Objective:**

The Radiotoxicity Bladder Biomarkers study aims to investigate the correlation between blood and urinary biomarkers and the intensity of acute RC symptoms and QoL in patients undergoing localized prostate cancer radiotherapy.

**Methods:**

This study included patients with low- or intermediate-risk localized prostate cancer who were eligible for localized radiotherapy. Blood and urinary biomarkers were analyzed before radiotherapy was initiated and at weeks 4 and 12 of radiation therapy. Patients completed questionnaires related to RC symptoms and QoL (International Prostate Symptom Score and Functional Assessment of Cancer Therapy-Prostate [FACT-P]) using a digital remote monitoring platform. The information was processed by means of an algorithm, which classified patients according to the severity of symptoms and adverse events reported. Levels of blood and urinary biomarkers were tested with the severity of acute RC symptoms and patient-reported QoL.

**Results:**

A total of 401 adverse events questionnaires were collected over the duration of this study from 20 patients. The most frequently reported adverse events at week 4 were pollakiuria, constipation, and diarrhea. In comparison with baseline, the mean FACT-P score decreased at week 4. A significant increase in the proportion of M2 phenotype cells (CD206+, CD163+, CD204+) at W12 compared to W0 was observed. An increase in serum and urine levels of macrophage colony-stimulating factor (M-CSF), hepatocyte growth factor, and macrophagic inflammatory protein was observed at week 12 compared to baseline levels. Baseline serum and urine M-CSF concentrations showed a significant negative correlation with FACT-P scores at weeks 4 and 12 (*r*=−0.65, *P*=.04, and *r*=−0.76, *P*=.02, respectively).

**Conclusions:**

The Radiotoxicity Bladder Biomarkers study is the first to explore the overexpression of inflammatory proteins in blood and urine of patients with symptoms of acute RC. These preliminary findings suggest that serum and urine levels of hepatocyte growth factor, M-CSF, and macrophagic inflammatory protein, as well as macrophage polarization, are mobilized after prostate radiotherapy. The elevated M-CSF levels in serum and urine at baseline were associated with the deterioration of QoL during radiotherapy. The results of this study may help to develop mitigation strategies to limit radiation damage to the bladder.

## Introduction

Prostate cancer is the most commonly diagnosed cancer among men in France, with 50,400 new cases and 8100 deaths in 2018 [1]. Between 2010 and 2018, improved diagnostic strategies and therapeutic management led to a 3.7% reduction in mortality while the survival rate has increased to 93% at 5 years and 80% at 10 years [1,2]. However, treatment-related adverse events can be serious and have an impact on compliance with treatment, frequency of hospitalization, and associated costs, as well as on patients’ quality of life (QoL) [3].

Radiation therapy (including conventional radiation therapy, stereotactic body radiation therapy, and brachytherapy) is an important therapeutic technique in the management of pelvic cancers, including prostate cancer [4-9]. Despite improvements in radiation techniques, pelvic radiotherapy is nonetheless associated with potential acute and late adverse events involving the bladder, which are collectively described referred to as radiation cystitis (RC). Though most treatment-related urinary events occurring after radiotherapy are of low grade, some patients may still present with severe symptoms of RC [10].

Early symptoms of RC include those which occur during treatment and up to 3 months after the cessation of radiotherapy, with an estimated all-grade incidence of nearly 50% after pelvic irradiation [10]. These side effects are characterized by frequent and urgent urination day and night, irritative symptoms, or pain. Obstructive symptoms or less hematuria may also be present [11]. In 5% to 10% of cases, complications appear later, more than 6 months after radiotherapy, whether or not they were preceded by early signs [10,12,13]. Such late-onset adverse events involve blood vessel damage and fibrosis of the bladder wall, which may progress chronically and lead to bladder atrophy and even retraction in the most extreme cases [10]. The clinical signs vary depending on the dominant clinical form: cystalgia, pollakiuria, bladder hyperactivity, or isolated mictional disorders. Classic clinical features dominate with recurrent and abundant hematuria, of variable frequency, which may even result in urinary retention with bladder clotting. The chronic and recurrent nature of hemorrhagic cystitis often has a considerable impact on patients’ QoL. The most severe forms, with clot formation and acute urinary retention, can be life threatening [10,13].

Immunity plays an important role in radiation-induced toxicity or inflammation [14,15]. During the repair process of radiation-induced injuries, inflammatory cells (macrophages, neutrophils, or lymphocytes) are recruited to the site of injury. Late inflammatory tissue diseases may develop through a continuous mechanism involving inflammation, hypoxia, and fibrosis [16]. The balance between M1 and M2 macrophages plays a central role in the fibrotic process, with a polarization toward M1 macrophages [17,18]. Moreover, functional tests measuring the apoptosis of CD4+ and CD8+ T lymphocytes after irradiation have demonstrated a significant association between these apoptotic lymphocytes and the risk of occurrence of late genitourinary toxicity [19].

The characteristics of interstitial cystitis are similar to RC both in terms of collagen accumulation and symptoms. Patients with interstitial cystitis have very severe genitourinary pain, and many are diagnosed as depressed and anxious. A positive correlation has been reported between elevated proinflammatory cytokines (IL-4 and macrophage-derived chemokines) in urine and the severity of interstitial cystitis [20,21].

The pathophysiology of RC thus remains poorly studied and not well understood. A number of factors have been identified, such as the dose of radiation, fractionation, and comorbidities (diabetes or tobacco smoking), but the risks of complications arising from access to bladder tissue postirradiation limits our knowledge and ability to develop therapies targeting this process [22,23]. It is essential to gain a better understanding of RC from the acute phase onward. This would help ensure the antitumor therapeutic efficacy of irradiation while minimizing undesirable effects on healthy tissue, particularly in the bladder. The identification of serum and urine biomarkers linked to RC is essential in order to characterize the kinetics of RC onset and predict the toxic effects of irradiation. This clinical trial thus aims to combine patient-related outcomes on adverse events and QoL following radiotherapy, with an analysis of serum and urinary biomarkers that may be predictive of toxicity.

The main objective of the Radiotoxicity Bladder Biomarkers (RABBIO) study is to identify markers of the inflammatory and remodeling processes involved in the occurrence of early (<3 months) RC in patients with localized prostate cancer.

## Methods

### Study Design

The RABBIO study is an observational, prospective, single-arm, exploratory study to identify factors potentially related to radiation-induced bladder toxicity in patients treated with radiotherapy for localized prostate cancer. This study was carried out at Bégin Military Hospital and Institut de Recherche Biomédicale des Armées. All eligible patients going through the hospital were presented the information about this study and were given the opportunity to participate upon consent.

### Ethical Considerations

This study was validated by the national ethics committees (IDRCB: 2021-A03196-35; favorable opinion of the South Mediterranean Committee for the Protection of Persons I February 3, 2022) and the French Data Protection Agency and was registered on ClinicalTrials.gov (NCT05246774). The survey complied with the principles set out in the Declaration of Helsinki. All patients were informed that the data collected may be used for research purposes and have given their written consent. The full, nonanonymized study data are only available to the investigator, and its storage in treatment follows the French regulations. In particular, the data are deidentified before it is used for analysis.

### Patient Population

The eligibility criteria for the RABBIO trial are listed in Textbox 1. As this study was exploratory, the sample size was not based on statistical reasoning. The variability and evolution of biomarkers over time and the history of the disease were not known. We hypothesized that about half the patients included will develop cystitis. In order to explore the links between biomarkers and the occurrence of RC, a sample size of 20 participants seemed acceptable [24,25].

Textbox 1. **Inclusion and exclusion criteria.**

**Inclusion criteria. Patients eligible for inclusion in Radiotoxicity Bladder Biomarkers (RABBIO) study must meet all of the following criteria:**

- Collection of signed informed consent form prior to participation in this study.
- Patient aged ≥18 years at the time of selection.
- Histologically confirmed adenocarcinoma of the prostate.
- Localized adenocarcinoma of the prostate according to the D’Amico classification.
- Eligible for external radiotherapy or brachytherapy.
- Patient affiliated to a social security scheme.
- Patient able to communicate well, understand, and comply with the requirements of this study according to the physician-investigator.
- Patient with a smartphone or computer to use the Cureety platform.

**Exclusion criteria. Patients meeting any of the following criteria are not eligible for inclusion in RABBIO study:**

- Patients with advanced or metastatic prostate cancer.
- Patients receiving preirradiation hormone therapy.
- Patients with bladder or urethral cancer or a history of cancer.
- Previous urinary tract surgery (bladder augmentation or cystectomy).
- Patient participating in an interventional clinical study.
- Patient with a history of pelvic irradiation.

### Participants’ Calendar

Early symptoms of RC are likely to occur during treatment or within 3 months of radiotherapy in about half of the patients. Therefore, the early manifestations of radiation-induced bladder toxicity were monitored for 3 months (W1 to W12) in order to identify biomarkers that could be related to the symptoms of acute RC.

**Figure 1.**
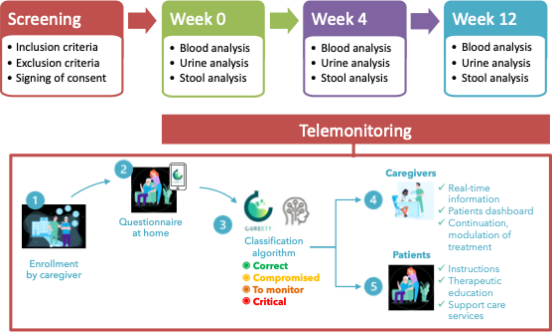
The RABBIO study design. RABBIO: Radiotoxicity Bladder Biomarkers.

The RABBIO study design is shown in Figure 1.

### Data Collection

#### Clinical Data

##### Demographics and Disease Characteristics

Patients’ demographic data and cancer characteristics (localized or biologically relapsed prostate cancer, stage of disease, radiation regimen, concomitant treatments, and comorbidities) were collected.

##### Clinical Examination

The clinical examination at each visit included performance index (performance status), weight, blood pressure, heart rate, and oxygen saturation.

##### Collection of Patient-Reported Outcomes Using the Cureety App

All questionnaires were completed by the patients in a digital form using the Cureety application. The various questionnaires and outcomes are detailed in the following sections: *Adverse Events*, *International Prostate Symptom Score*, and *Functional Assessment of Cancer Therapy-Prostate*.

##### Adverse Events

Remote monitoring of urinary symptoms reported by patients was ensured by means of the Cureety platform [26] according to the Common Terminology Criteria for Adverse Events. Patients completed the Pelvic Radiation Adverse Events Questionnaire at the inclusion visit, then once a week for 3 months (wk 1 to wk 12). The questionnaire includes 15 items on fatigue, nausea or vomiting, pain, hematuria, frequency of urination (pollakiuria), urinary burning, diarrhea, fecal incontinence, urinary leakage, blood in the stool (rectorrhagia), constipation, weight loss, and dysuria.

##### Clinical Classification and Remote Patient Monitoring With the Cureety Platform

Using the data from the adverse events questionnaires, the *conformité européenne*–marked Cureety TechCare algorithm classified patients into 1 of 4 states [26]:

- Normal or minor (green)
- Fragile (yellow)
- At risk (Orange)
- Critical (Red)

Each patient received therapeutic advice depending on the severity of the symptoms. If the patient’s condition changes to orange (at risk) or red (critical), rapid management of the patient was initiated by the health care team (Figure 1).

##### International Prostate Symptom Score

The International Prostate Symptom Score (IPSS) is a structured and validated self-report questionnaire that assesses lower urinary tract voiding disorders. The questions cover the following items: incomplete emptying of the bladder, frequency of micturition, intermittent micturition (stopping and restarting the stream), urgent micturition (feeling of “urgency”), weak stream, effort to urinate (forcing or pushing), and nocturia.

The total of the 7 items gives the international score for prostate symptoms in terms of severity. Each question has a score from 1 to 5, for a total of 35 points maximum:

- Score of 0‐7: no or mild symptoms
- Score of 8‐19: moderate symptoms
- Score of 20‐35: severe symptoms

##### Functional Assessment of Cancer Therapy-Prostate

The Functional Assessment of Cancer Therapy-Prostate (FACT-P) is a prostate cancer-specific self-report questionnaire that assesses weight loss, appetite, pain, physical comfort, urinary, sexual and bowel function in 12 items. The score ranges from 0 to 156, with higher scores reflecting better QoL.

The IPSS, FACT-P were completed by each patient via the Cureety platform at inclusion and in the course of visits at weeks 4 and 12.

### Biological Data Collection

#### Biological Biomarkers

The variation in expression of major biomarkers reported in the literature, including both serum inflammatory and remodelling biomarkers as well as urine biomarkers, was assessed at baseline, week 4, and week 12. Further, 6 mL of blood and 5 mL of urine per patient or visit were used for the analysis. The biomarkers measured with these methods were as follows:

- Biomarkers related to inflammation: macrophage migration inhibitory factor; cytokines IL-1α, IL-1β, IL-4, IL-6, IL-7, IL-8, IL-10, IL-13, IL-17α; macrophagic inflammatory protein (MIP-1α); tumor necrosis factor (TNFα); vascular cell adhesion molecule-1 (VCAM-1); intercellular adhesion molecule-1; chemotactic cytokines (MCP-1, MCP-3, RANTES); C-X-C chemokine motif (CXCL10); M1/M2 ratio; CD4+ and CD8+ T lymphocytes; and C-reactive protein.
- Biomarkers of remodeling: plasminogen activator inhibitor 1 (PAI-1), metalloproteinases (MMP-9), matrix metalloproteinase inhibitors (TIMP1 and TIMP2); hepatocyte growth factor (HGF), placental growth factor, vascular endothelial growth factor, epidermal growth factor, heparin-binding epidermal growth factor, nerve tissue growth factor, and GP51 glycoprotein.

The variation in expression of circulating markers was analyzed by means of the MILLIPLEX MAP (Multi-Analyte Profiling) technique, using Luminex xMAP technology assessed on the principle of Enzyme-Linked Immunosorbent Assay (Table S1 in Multimedia Appendix 1).

#### Protocol for Analysis of the Circulating Immune Population by Flow Cytometry

Analysis of the immune cell population by flow cytometry was performed at baseline, week 4, and week 12 after the start of irradiation.

### Statistical Analysis

Patients’ characteristics were compared using chi-square and Student 1-tailed Student *t* tests. Correlations between patients’ characteristics, tumor characteristics, treatment toxicities, and blood and urinary biological parameters were assessed using the Pearson correlation test. Follow-up was scheduled at weeks 4 and 12.

Basic statistics were used for continuous variables, missing n (if applicable), mean, type of deviation, median, first and third quartile (Q1 and Q3), and minimum and maximum. Frequency and percentage were used for categorical variables. A Mann-Whitney *U* test was used to compare groups for nonparametric variables, based on the data distribution.

The type I error (α) was 5% (two-sided), and type II error (β) was 20%, that is, a power (1 – β) of 80%.

These statistical analyses were carried out with SAS (version 9.4; SAS Institute Inc) and R (R Foundation for Statistical Computing) [27].

## Results

### Patient Population

From March 2022 to January 2023, a total of 20 patients were included in our study. The median age was 76 (IQR 65-89) years. Of these, 65% (n=13) had at least one comorbidity, and 35% (n=7) had type 2 diabetes mellitus. All patients had localized disease. Seventeen patients (80%) had de novo localized prostate cancer and 3 (15%) had biochemical recurrence without metastases. The median Gleason score was 7 (IQR 6‐7). The median prostate specific antigen was 7.85 (IQR 0.27‐35). Per the inclusion criteria, all patients had a performing status in the 0‐1 range.

The median dose to the prostate was 60 (IQR 60‐78) Gy. Twelve patients received 60 Gy in 20 fractions. The other 8 received 78 Gy.

A total of 112 blood and urine samples were collected.

Compliance with the digital platform was 100% at baseline, 93% at W4, and 100% at W12. Patients’ baseline characteristics are summarized in Table 1.

**Table 1.** Baseline patients’ characteristics.

Variable		Values
Number of patients, n (%)		20 (100)
Age (years), median (IQR)		73 (63‐89)
Prostate-specific antigen (ng/mL), median (IQR)		7.85 (0.27‐35)
Gleason score, median (IQR)		7 (6-7)
Tumor stage, n (%)		
	T1c	4 (20)
	T2	2 (10)
	T2a	1 (5)
	T2b	1 (5)
	T2c	2 (10)
	pT3R1	1 (5)
	Tx	9 (45)
N0 (no nodes metastasized), n (%)		20 (100)
M0 (no metastasis), n (%)		20 (100)
Comorbidities, n (%)		
	Cardiac (yes)	13 (65)
	Diabetes (yes)	7 (35)
Localized prostate cancer de novo, n (%)		17 (85)
Biochemical recurrence, n (%)		3 (15)
Dose prostate delivered (Gy), median (IQR)		60 (60‐78)

### Clinical Data

**Figure 2.**
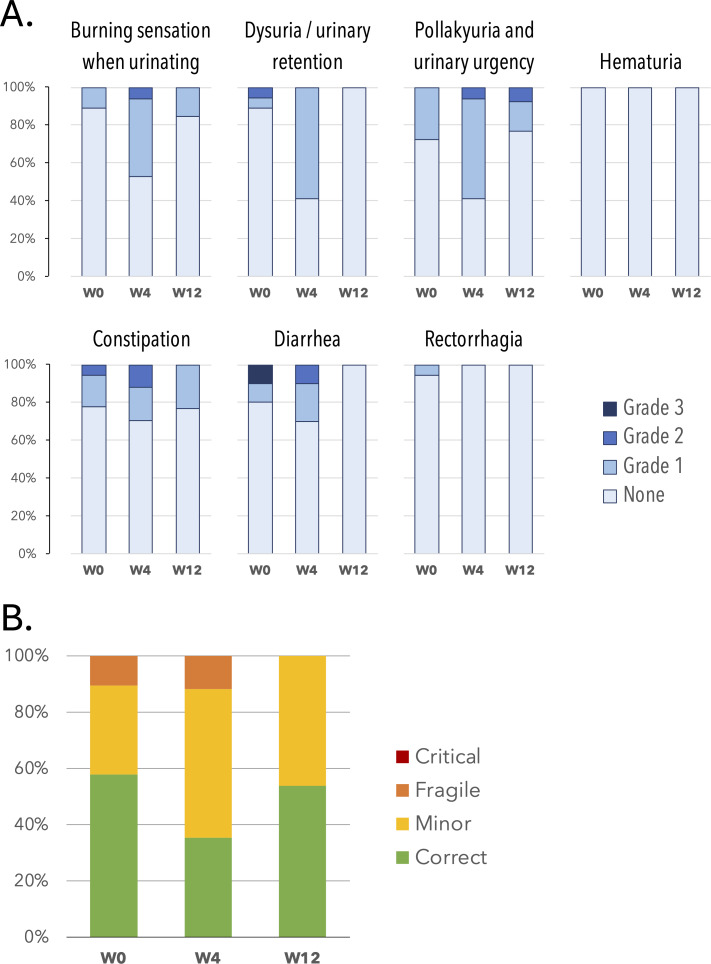
(A) Adverse events W0 to W12. (B) Clinical classifications W0 to W12. The clinical classifications were determined by the software medical device Cureety TechCare (scoring from the combination of adverse events). W: week.

To date, we have collected a total of 401 adverse event questionnaires over the duration of this study. Patients reported the largest number of adverse events at week 4 (Figure 2A), at which point the associated clinical classifications also indicated a worsened health state (Figure 2B).

The most frequently reported adverse events at week 4 were pollakiuria (10/17 grade 1 or 2, 58.8%), constipation (5/17 grade 1 or 2, 29%), and diarrhea (6/18 grade 1 or 2, 33%; Figure 2A). At week 4, 53% (9/17) of the clinical classifications of the patients were evaluated as “minor” and 12% (2/17) as “fragile” (Figure 2B).

**Figure 3.**
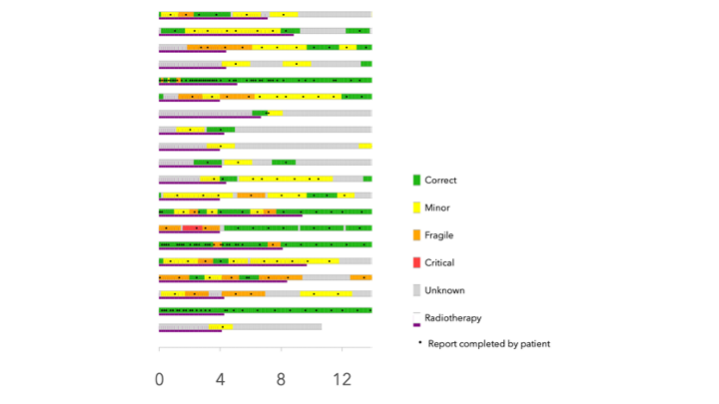
Timelines for each patient during irradiation: each line represents the monitoring of a patient and shows the clinical classifications computed by the device algorithm (green, yellow, orange, or red) from the completed questionnaires (black dots). The end of each timeline corresponds to the end of this study’s analysis.

In this study, patients monitoring was reported up to 12 weeks and Figure 3 displays patients’ tolerance in the form of a visual timeline showing the clinical classifications (green, yellow, orange, or red) over the monitoring period, including irradiation (indicated by a purple line under the timeline).

All patients with complications received symptomatic treatments adapted to the reported adverse events.

### IPSS and FACT-P Status

Patients were followed for a full year. IPSS was assessed at W0, W4, and W12 for all patients. At baseline, 60% of patients reported minor urinary symptoms. Symptoms were moderate for 50% of patients and severe for another 20% at W4. At W12, 80% of the patients reported minor symptoms.

**Figure 4.**
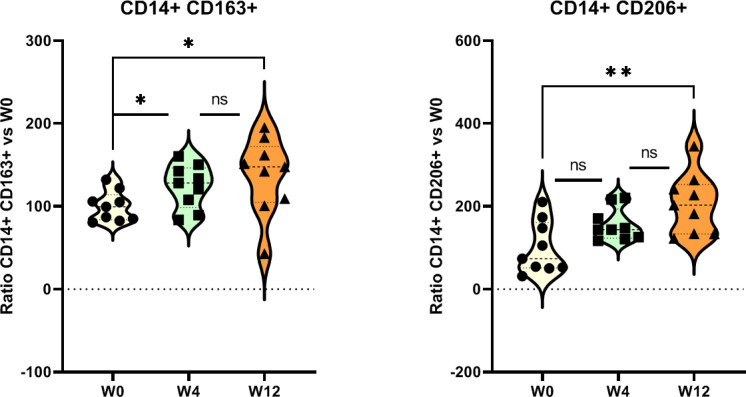
Quality of life measured via the FACT-P score. FACT-P: Functional Assessment of Cancer Therapy-Prostate.

Similarly, FACT-P was assessed for all patients at W0, W4, and W12. The mean FACT-P score at baseline for all patients was 34 (SD 24-40), which changed to 30 (SD 20-35) at W4, and 39 (SD 37-42) at W12. Evolution of FACT-P score before, during, and after irradiation is reported in Figure 4.

### Macrophage Polarization During Prostate Irradiation

We assessed the change in polarization of peripheral macrophages following irradiation. The results showed a significant increase in the proportion of M2 phenotype cells (CD206+, CD163+, and CD204+) at W12 compared to W0.

**Figure 5.**
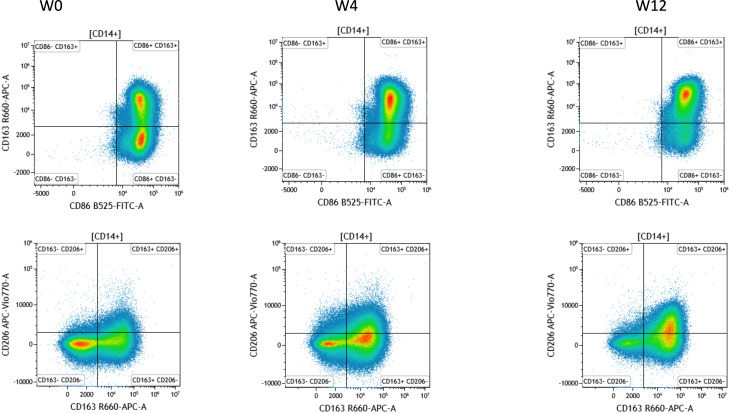
Analysis of the circulating immune cell population by flow cytometry. (A) Representative FACS dot plots to identify macrophage M2a (CD86+ CD163+) and M2c (CD163+ CD206+). (B) Increased ratio of macrophage phenotype M2 (macrophage M2a [CD86+ CD163+] and M2c [CD163+ CD206+]). Mann Whitney test: ratio versus W0 (before radiotherapy), *P<.05, **P<.01, ns. FACS: flow cytometry; ns: not significant; W: week.

A significant decrease in the proportion of M1 phenotype cells (CD86+) was observed at W4 following irradiation (Figure 5).

### Changes in Serum Cytokine Levels

A total of 180 blood samples were taken before the start of treatment, and then at W4 and W12.

**Figure 6.**
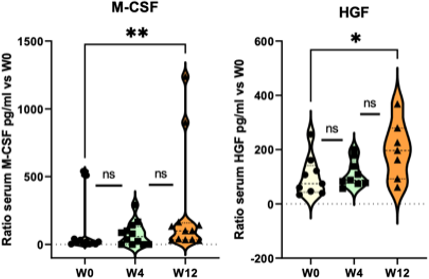
Changes in serum cytokine levels. Mann Whitney test: ratio versus W0 (before radiotherapy), *P<.05, **P<.01, ns. HGF: hepatocyte growth factor; M-CSF: macrophage colony-stimulating factor; ns: not significant; W: week.

Serum HGF levels in patients with prostate cancer were found to be significantly higher at W12 than before radiotherapy (*P*<.001; Figure 6).

Among the inflammatory proteins measured, a significant increase in serum macrophage colony-stimulating factor (M-CSF) levels was also observed at W12 compared to levels determined before radiotherapy (*P*<.001; Figure 6).

In our cohort, no significant increase in profibrotic proteins was observed during the acute phase.

### Changes in Urine Cytokine Levels

To investigate possible changes in cytokine profiles during irradiation, the concentrations of 33 proteins were measured in patients’ urine before radiotherapy treatment initiation, and again at W4 and W12.

**Figure 7.**
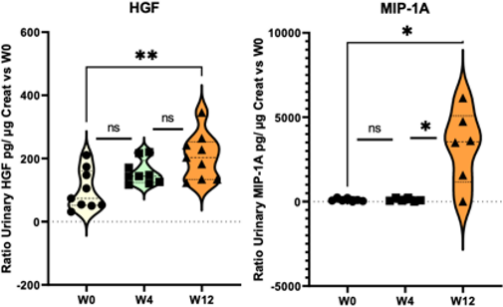
Changes in urine cytokine levels. Mann Whitney test: ratio versus W0 (before radiotherapy), *P<.05, **P<.01, ns. HGF: hepatocyte growth factor; MIP: macrophagic inflammatory protein; ns: not significant; W: week.

Among the inflammatory proteins measured, a significant increase in urine MIP-1A and HGF levels was found at week 12 compared to baseline (*P*<.001, Figure 7).

### Correlation Between Genitourinary Toxicity Grade and HGF, SHBG, and IL8 Urine Concentrations

**Figure 8.**
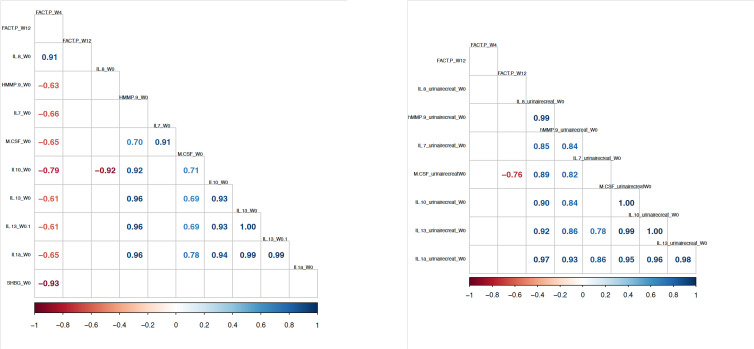
Correlation matrices between FACT-P scores and cytokine concentrations in serum (**A**) and urine (**B**). Empty cells indicate a nonsignificant correlation (*P*>.05). When significant (*P*<.05), numbers correspond to *r* correlation coefficients, positive or negative (Pearson test). FACT-P: Functional Assessment of Cancer Therapy-Prostate.

Possible correlations between maximum acute genitourinary toxicity grade and serum and urine concentrations in patients with prostate cancer treated with radiotherapy are presented in Figure 8.

Significant negative correlations with FACT-P scores were found at week 4 with respect to baseline serum M-CSF concentrations (r=−0.65, *P*=.04), and at week 12 with respect to baseline urine M-CSF concentrations (*r*=−0.76, *P*=.02).

## Discussion

### Principal Findings

This prospective study is the first to explore the overexpression of inflammatory proteins in the blood and urine of patients with symptoms of acute RC and to assess the correlation between electronic patient‐reported outcomes and biomarkers. Our principal findings include (1) significant overexpression of inflammatory proteins such as M2 macrophages, HGF, M-CSF, and MIP-1 in patients with RC, suggesting their involvement in the pathophysiology of the condition, and (2) a demonstrated correlation between higher levels of urinary M-CSF and increased bladder toxicity, indicating that urinary M-CSF could serve as a predictive biomarker for radiation-induced bladder damage. These results provide new insights into the molecular mechanisms underlying RC and highlight potential biomarkers for the early detection and management of this condition.

Radiotherapy is a powerful tool in the management of localized prostate cancer. Hamdy et al [28] reported the results of the PROTECT study after 15 years of follow-up. This study assessed the effectiveness of conventional treatments in clinically localized prostate cancer. A total of 545 patients underwent radiotherapy. After median follow-up of 15 years, 16 (2.9%) patients had died of prostate cancer in the radiotherapy group. No significant difference in prostate cancer mortality was found between the trial groups (monitoring, surgery, or radiotherapy; *P*=.53). This study confirmed the efficacy of radiation in the management of localized prostate cancer.

However, the incidence of RC is stable over time for all types of pelvic irradiation techniques. In the randomized phase 3 multicenter HYPRO trial, the cumulative incidence by 120 days after radiotherapy of grade 2 or worse acute genitourinary toxicity was 58% (95% CI 52.9% to 62.7%) in the standard fractionation group versus 60.5% (95% CI 55.8 to 65.3) in the hypofractionation group, a difference of 3% (95% CI −2.99% to 8.48%; odds ratio 1.12, 95% CI 0.84 to 1.49, *P*=.43). Approximately 22% of the patients reported grade 2 or worse genitourinary toxicity, and 2 patients (<1%) reported grade toxicity 4 in the 3 months after irradiation [29]. Dearnaley et al [30] also reported that more than 40% of patients presented Radiation Therapy Oncology Group grade 2 or worse bladder toxicity while acute Radiation Therapy Oncology Group bladder symptoms peaked at 4‐5 weeks in hypofractionated radiation schedules.

Moreover, this incidence is possibly underestimated considering the discrepancy between the clinician’s description of the severity of the symptom and the patient’s experience [31]. In a study assessing QoL and satisfaction with outcome in prostate cancer survivors, Sanda et al [32] reported that urinary symptoms had a significant impact on their QoL at 2 months after irradiation. In total, 30% of patients in the External Beam Radiation Therapy arm and 39% of patients in the brachytherapy arm reported urinary discomfort. Patients in the brachytherapy arm reported a significant decrease in urinary irritation or obstruction and incontinence compared to baseline (*P*<.001). At one year, 18% of patients in the brachytherapy group and 11% in the External Beam Radiation Therapy group reported moderate or worse distress related to overall urinary symptoms. Incontinence after brachytherapy was reported by 4%‐6% of patients 1 to 2 years after treatment, and was significantly related to worse QoL [32].

As a first step, our work reported patients’ experience during radiotherapy through telemonitoring, in order to have a picture as close as possible to reality and assess the impact of side effects on their QoL. Collecting data from patients (patient-reported outcomes) helps to correct the discrepancy in the severity of the side effects when reported by the clinician or by the patient [31]. Our results confirm the impact of urinary symptoms on patients’ QoL, which deteriorated at W4 due to an increase in urinary symptoms, with 53% classified as “minor” and 12% as “fragile.” The main symptoms were related to pollakiuria.

The second step involved analyzing the pathophysiology of acute RC, which is often the initial stage of late RC lesions with a risk of life-threatening chronic hemorrhagic cystitis. Our findings reveal an early polarization of M2 phenotype macrophages as early as W4 with a significant increase at W12.

Macrophages are immune cells that play a crucial role in the repair and remodeling of tissues after injury by infiltrating the irradiated area and releasing various factors that promote tissue repair and fibrosis. Macrophages can be classified into two main subsets: proinflammatory M1 macrophages, which are associated with tissue damage, and anti-inflammatory M2 macrophages which are involved in tissue repair and remodeling [33]. The balance between these two phenotypes ensures homeostasis, with M2 macrophages apparently involved in the pathophysiology of fibrosis and radiotoxicity [34].

The M2 macrophages produce a range of cytokines and growth factors, such as transforming growth factor-beta (TGF-β), platelet-derived growth factor, and fibroblast growth factor, which stimulate the proliferation and differentiation of fibroblasts and the deposition of extracellular matrix (ECM) proteins [34,35]. M2 macrophages have been shown to play a critical role in the initiation and progression of fibrotic diseases in various organs, including the liver, lung, and kidney [35-38]. M2 macrophages also inhibit the activity of proinflammatory T cells and promote the recruitment and activation of regulatory T cells, resulting in a shift toward an anti-inflammatory environment that favors fibrosis [39]. Furthermore, M2 macrophages can interact with other cell types, such as myofibroblasts and endothelial cells, to promote fibrogenesis [34,40]. The polarization of macrophages toward the M2 phenotype has been shown to play a critical role in the development of fibrosis following radiation-induced tissue damage. Irradiation has been shown to induce the recruitment of M2 macrophages which release various factors, such as TGF-β and platelet-derived growth factor. These factors promote the differentiation and activation of fibroblasts, which are the primary cells responsible for the production and deposition of ECM components, such as collagen and fibronectin, that form the fibrotic scar tissue [18,41].

The role of M2 macrophages in the development of radiation-induced fibrosis in various organs, including the lung, liver, and skin has been investigated in several studies. In a mouse model of radiation-induced lung fibrosis (RILF), the recruitment of M2 macrophages to the lung was found to be associated with the development of fibrosis, while depletion of macrophages or inhibition of M2 polarization reduced the extent of fibrosis [42,43]. Similarly, in a rat model of radiation-induced liver fibrosis, M2 macrophages were found to be the primary source of TGF-β, which promoted the differentiation of hepatic stellate cells into myofibroblasts, leading to the development of fibrosis [44]. In addition to promoting the differentiation and activation of fibroblasts, M2 macrophages can also contribute directly to the development of fibrosis by producing ECM components, such as collagen. In a study of radiation-induced skin fibrosis, M2 macrophages were shown to be a significant source of collagen in the irradiated skin, while depletion of macrophages or inhibition of M2 polarization reduced collagen deposition and the extent of fibrosis [34]. An early and maintained polarization of macrophages into the M2 phenotype could therefore be involved in the development of acute and late RC.

Second, we investigated blood and urine biomarkers. A significant irradiation-induced increase in HGF was observed in blood and urine. HGF is a pleiotropic cytokine implicated in various physiological and pathological processes, including tissue repair and fibrosis. HGF is a potent stimulator of epithelial cell growth, migration, and survival, and plays an important role in the regeneration and repair of various organs, including the liver, kidney, and lung [45]. Nonetheless, an elevated and persistent level is also involved in the pathophysiology of radiation-induced toxicity. Zwaans et al [14] analyzed urine samples from prostate cancer survivors who had undergone radiation therapy to identify changes in excreted urinary proteins involved in fibrosis, inflammation, and vascular biology. They reported that HGF concentration was significantly higher in patients with high symptom scores and positively associated with hematuria and a diagnosis of RC [14]. In our study, we have demonstrated that HGF secretion is induced by radiotherapy with a significant increase at W12. Initially this protein is involved in the repair process but over time continued secretion leads to the permanent recruitment of M2 macrophages and thus to the development of chronic RC.

Third, we observed a significant increase in urine levels of MIP-1α. MIP-1α, also known as CCL3, is a chemokine involved in the recruitment and activation of immune cells, including macrophages and T cells, in response to tissue injury or inflammation [46]. Recent studies have shown that MIP-1α may also play a role in the development of fibrosis. Heinrichs et al [26] demonstrated that MIP-1α promoted liver fibrosis in a mouse model, by recruiting immune cells. Deletion of MIP-1α reduced liver fibrosis [26]. Yang et al [47] reported that thoracic irradiation in in vitro and in vivo models increased MIP-1α levels, which was linked to inflammation and fibrosis, whereas irradiated mice lacking MIP-1α or its receptor, CCR1, did not develop lung inflammation or fibrosis [47].

Finally, our findings suggest that M-CSF levels could serve as a valuable prognostic factor for RC. M-CSF or colony stimulating factor 1 is a cytokine that plays an important role in the regulation of the immune system and tissue repair, more specifically in the differentiation, proliferation, and survival of monocytes and macrophages, and is critical for the maintenance of tissue homeostasis. M-CSF has also been linked to the pathogenesis of radiation-induced fibrosis and radiation toxicity. Baran et al [48] investigated the role of M-CSF in the pathogenesis of pulmonary fibrosis in a mouse model and human patients. They reported that patients with idiopathic pulmonary fibrosis had elevated levels of M-CSF in bronchoalveolar lavage fluid compared to normal volunteers. On the other hand, M-CSF-/- mice were protected from bleomycin-induced pulmonary fibrosis [48]. Meziani et al [43] reported an accumulation of pulmonary macrophages, particularly M2 macrophages, in RILF. Blocking the interaction between M-CSF and its receptor, however, leads to a depletion of M2 macrophages and blocks the development of RILF [43]. Kopčalić et al [23] reported a correlation between TGF-β1 and genitourinary toxicity in localized or locally advanced patients with prostate cancer treated with radiotherapy [23]. Although no such correlation was observed in our study, it is consistent with our findings concerning the polarization of macrophages toward the M2 phenotype which are responsible for TGF-β1 secretion.

Notwithstanding the promising results of the RABBIO study, several limitations should be taken into consideration. First, this study was conducted on a relatively small number of patients that may limit the generalizability of our findings. Second, this study is restricted to patients with intermediate-risk localized prostate cancer undergoing localized radiotherapy limiting its applicability to other pathological settings, such as the association of radiotherapy with hormonotherapy known to alter immunity. Despite these limitations, our study provides valuable insights into irradiation inducing immune changes and may inform on the development of future interventions to improve QoL for patients undergoing radiation therapy. We need to confirm these results in an independent validating cohort.

Our results reveal that pelvic irradiation for prostate cancer increases the secretion of HGF, M-SCF, and MIP-1α which act synergistically to induce macrophage polarization into the M2 phenotype, possibly favoring bladder toxicity and fibrosis. Inhibition of these molecules and in particular of M-CSF in patients with high levels could be taken as a therapeutic approach to prevent or mitigate RC incidence.

### Conclusion

This prospective study is the first to explore the overexpression of inflammatory proteins in the blood and urine of patients with symptoms of acute RC. Our first results suggest a central role of serum and urine HGF, M-CSF, MIP-1α, and macrophage polarization in the pathophysiology of RC. Moreover, an elevated level of M-CSF in serum and urine at baseline was found to be associated in the deterioration of QoL for localized patients with prostate cancer during radiotherapy.

Though cystitis can have significant implications for the QoL of affected patients, there is currently no standard established to identify patients at risk. There is a need for more sensitive and specific markers. In this study, we looked at an extended set of biomarkers as potential indicators of RC. These markers offer an opportunity for significant improvement in the early detection and management of cystitis, which could help improve diagnostic accuracy, identify at-risk patients earlier, and implement preventive management strategies.

At present, the lack of in-depth discussion of therapeutic management, hospitalizations, and costs in relation to reported symptoms and the potential link with biological markers is a major limitation of this study. However, it is essential to stress that these complex and interconnected aspects require a detailed analysis that would go beyond the scope of the present investigation. These crucial elements will be addressed in future work dedicated specifically to the clinical management of identified cases, highlighting therapeutic implications, hospitalization requirements, and associated financial considerations. In-depth analysis of these aspects will contribute to a more comprehensive understanding of the disease, enabling more effective patient management. By focusing on quality of care, optimization of treatment protocols, and efficient management of medical resources, future work will aim to provide practical, informed recommendations for health care professionals and policy makers. In summary, although these issues were not addressed in this study, they represent a promising area of research that will be explored in depth in our future work.

The results of this study may allow us to develop strategies to limit radiation damage and improve patients’ QoL, as well as predictive or prognostic models of bladder toxicity from irradiation radiotherapy in patients with prostate cancer combining clinical parameters, individual patient characteristics, and M-CSF levels in urine and blood.

## Supplementary material

10.2196/48225 Multimedia Appendix 1 Supplementary materials regarding flow cytometry and assays.

## Abbreviations

ECM: extracellular matrix
FACT-P: Functional Assessment of Cancer Therapy-Prostate
HGF: hepatocyte growth factor
IPSS: International Prostate Symptom Score
M-CSF: macrophage colony-stimulating factor
MAP: Multi-Analyte Profiling
MIP: macrophagic inflammatory protein
QoL: quality of life
RABBIO: Radiotoxicity Bladder Biomarkers
RC: radiation cystitis
RILF: radiation-induced lung fibrosis
TGF-β: transforming growth factor-beta

## References

[1] Le cancer de la prostate - les cancers les plus fréquents. Intitut National du Cancer.

[2] Siegel RL, Miller KD, Wagle NS, Jemal A (2023). Cancer statistics, 2023. CA A Cancer J Clin.

[3] Taylor JM, Chen VE, Miller RC, Greenberger BA (2020). The impact of prostate cancer treatment on quality of life: a narrative review with a focus on randomized data. Res Rep Urol.

[4] Zaorsky NG, Harrison AS, Trabulsi EJ (2013). Evolution of advanced technologies in prostate cancer radiotherapy. Nat Rev Urol.

[5] Daly T (2020). Evolution of definitive external beam radiation therapy in the treatment of prostate cancer. World J Urol.

[6] Kamran SC, D’Amico AV (2020). Radiation therapy for prostate cancer. Hematol Oncol Clin North Am.

[7] Tan TJ, Siva S, Foroudi F, Gill S (2014). Stereotactic body radiotherapy for primary prostate cancer: a systematic review. J Med Imaging Radiat Oncol.

[8] Zaorsky NG, Davis BJ, Nguyen PL (2017). The evolution of brachytherapy for prostate cancer. Nat Rev Urol.

[9] Incrocci L, Wortel RC, Alemayehu WG (2016). Hypofractionated versus conventionally fractionated radiotherapy for patients with localised prostate cancer (HYPRO): final efficacy results from a randomised, multicentre, open-label, phase 3 trial. Lancet Oncol.

[10] Helissey C, Cavallero S, Brossard C, Dusaud M, Chargari C, François S (2020). Chronic inflammation and radiation-induced cystitis: molecular background and therapeutic perspectives. Cells.

[11] Rigaud J, Le Normand L, Labat JJ (2010). Cystite radique. Pelv Perineol.

[12] Zelefsky MJ, Levin EJ, Hunt M (2008). Incidence of late rectal and urinary toxicities after three-dimensional conformal radiotherapy and intensity-modulated radiotherapy for localized prostate cancer. Int J Radiat Oncol Biol Phys.

[13] Goucher G, Saad F, Lukka H, Kapoor A (2019). Canadian Urological Association best practice report: diagnosis and management of radiation-induced hemorrhagic cystitis. Can Urol Assoc J.

[14] Zwaans BMM, Nicolai HE, Chancellor MB, Lamb LE (2020). Prostate cancer survivors with symptoms of radiation cystitis have elevated fibrotic and vascular proteins in urine. PLoS One.

[15] Shi X, Shiao SL (2018). The role of macrophage phenotype in regulating the response to radiation therapy. Transl Res.

[16] Chargari C, Supiot S, Hennequin C, Chapel A, Simon JM (2020). Treatment of radiation-induced late effects: what’s new?. Cancer Radiot.

[17] Mukherjee D, Coates PJ, Lorimore SA, Wright EG (2014). Responses to ionizing radiation mediated by inflammatory mechanisms. J Pathol.

[18] Meziani L, Deutsch E, Mondini M (2018). Macrophages in radiation injury: a new therapeutic target. Oncoimmunology.

[19] Foro P, Algara M, Lozano J (2014). Relationship between radiation-induced apoptosis of T lymphocytes and chronic toxicity in patients with prostate cancer treated by radiation therapy: a prospective study. Int J Radiat Oncol Biol Phys.

[20] Kim J, Kim WT, Kim WJ (2020). Advances in urinary biomarker discovery in urological research. Investig Clin Urol.

[21] Vera PL, Preston DM, Moldwin RM (2018). Elevated urine levels of macrophage migration inhibitory factor in inflammatory bladder conditions: a potential biomarker for a subgroup of interstitial cystitis/bladder pain syndrome patients. Urology.

[22] Stankovic V, Džamic Z, Pekmezovic T (2016). Acute and late genitourinary toxicity after 72 Gy of conventionally fractionated conformal radiotherapy for localised prostate cancer: impact of individual and clinical parameters. Clin Oncol.

[23] Kopčalić K, Matić IZ, Besu I (2022). Circulating levels of IL-6 and TGF-β1 in patients with prostate cancer undergoing radiotherapy: associations with acute radiotoxicity and fatigue symptoms. BMC Cancer.

[24] Hertzog MA (2008). Considerations in determining sample size for pilot studies. Res Nurs Health.

[25] Julious SA (2016). Pilot studies in clinical research. Stat Methods Med Res.

[26] Heinrichs D, Berres ML, Nellen A (2013). The chemokine CCL3 promotes experimental liver fibrosis in mice. PLoS One.

[27] The R Project for Statistical Computing. r-project.org.

[28] Hamdy FC, Donovan JL, Lane JA (2023). Fifteen-year outcomes after monitoring, surgery, or radiotherapy for prostate cancer. N Engl J Med.

[29] Aluwini S, Pos F, Schimmel E (2015). Hypofractionated versus conventionally fractionated radiotherapy for patients with prostate cancer (HYPRO): acute toxicity results from a randomised non-inferiority phase 3 trial. Lancet Oncol.

[30] Dearnaley D, Syndikus I, Mossop H (2016). Conventional versus hypofractionated high-dose intensity-modulated radiotherapy for prostate cancer: 5-year outcomes of the randomised, non-inferiority, phase 3 CHHiP trial. Lancet Oncol.

[31] Pakhomov SV, Jacobsen SJ, Chute CG, Roger VL (2008). Agreement between patient-reported symptoms and their documentation in the medical record. Am J Manag Care.

[32] Sanda MG, Dunn RL, Michalski J (2008). Quality of life and satisfaction with outcome among prostate-cancer survivors. N Engl J Med.

[33] Wynn TA (2008). Cellular and molecular mechanisms of fibrosis. J Pathol.

[34] Lis-López L, Bauset C, Seco-Cervera M, Cosín-Roger J (2021). Is the macrophage phenotype determinant for fibrosis development?. Biomedicines.

[35] Wang C, Ma C, Gong L (2021). Macrophage polarization and its role in liver disease. Front Immunol.

[36] Laskin DL, Malaviya R, Laskin JD (2019). Role of macrophages in acute lung injury and chronic fibrosis induced by pulmonary toxicants. Toxicol Sci.

[37] Braga TT, Correa-Costa M, Guise YFS (2012). MyD88 signaling pathway is involved in renal fibrosis by favoring a TH2 immune response and activating alternative M2 macrophages. Mol Med.

[38] Beljaars L, Schippers M, Reker-Smit C (2014). Hepatic localization of macrophage phenotypes during fibrogenesis and resolution of fibrosis in mice and humans. Front Immunol.

[39] Guan T, Zhou X, Zhou W, Lin H (2023). Regulatory T cell and macrophage crosstalk in acute lung injury: future perspectives. Cell Death Discov.

[40] Vasse GF, Nizamoglu M, Heijink IH (2021). Macrophage-stroma interactions in fibrosis: biochemical, biophysical, and cellular perspectives. J Pathol.

[41] Cytlak UM, Dyer DP, Honeychurch J, Williams KJ, Travis MA, Illidge TM (2022). Immunomodulation by radiotherapy in tumour control and normal tissue toxicity. Nat Rev Immunol.

[42] Duru N, Zhang Y, Gernapudi R (2016). Loss of miR-140 is a key risk factor for radiation-induced lung fibrosis through reprogramming fibroblasts and macrophages. Sci Rep.

[43] Meziani L, Mondini M, Petit B (2018). CSF1R inhibition prevents radiation pulmonary fibrosis by depletion of interstitial macrophages. Eur Respir J.

[44] Xi S, Zheng X, Li X (2021). Activated hepatic stellate cells induce infiltration and formation of CD163+ macrophages via CCL2/CCR2 pathway. Front Med.

[45] Nakamura T, Mizuno S (2010). The discovery of hepatocyte growth factor (HGF) and its significance for cell biology, life sciences and clinical medicine. Proc Jpn Acad Ser B Phys Biol Sci.

[46] Bhavsar I, Miller CS, Al-Sabbagh M (2015). Macrophage inflammatory protein-1 alpha (MIP-1 alpha)/CCL3: as a biomarker. Gen Methods Biomark Res Appl.

[47] Yang X, Walton W, Cook DN (2011). The chemokine, CCL3, and its receptor, CCR1, mediate thoracic radiation-induced pulmonary fibrosis. Am J Respir Cell Mol Biol.

[48] Baran CP, Opalek JM, McMaken S (2007). Important roles for macrophage colony-stimulating factor, CC chemokine ligand 2, and mononuclear phagocytes in the pathogenesis of pulmonary fibrosis. Am J Respir Crit Care Med.

