# Supplementary material for: Correlation Between Electronic Patient-Reported Outcomes and Biological Markers of Key Parameters in Acute Radiation Cystitis Among Patients With Prostate Cancer (RABBIO): Prospective Observational Study
Source: JMIR Cancer. 2024 Dec 12;10:e48225. doi: 10.2196/48225 (PMC11656992; doi:10.2196/48225)
Supplement: Multimedia Appendix 1 [file cancer-v10-e48225-s001.docx]

**Table S1.** MACS flow cytometry (Miltenyi Biotec).

| MACS Flow cytometry (Miltenyi Biotec) | Reference |
| --- | --- |
| MACS® Comp Bead Kit, anti-REA | 130-104-693 |
| MACS® Comp Bead Kit, anti-mouse Igκ | 130-097-900 |
| FcR Blocking Reagent, human | 130-059-901 |
| Viobility™ Fixable Dyes | 130-130-403 |
| CD14 Antibody, anti-human, PerCP-Vio® 700, REAfinity™ | 130-110-523 |
| CD45 Antibody, anti-human, VioGreen™, REAfinity™ | 130-110-638 |
| CD86 Antibody, anti-human, APC-Vio® 770, REAfinity™ | 130-116-163 |
| CD163 Antibody, anti-human, APC, REAfinity™ | 130-112-129 |
| CD197 (CCR7) Antibody, anti-human, PE, REAfinity™ | 130-117-396 |
| CD200R Antibody, anti-human, REAfinity™ | 130-111-290 |
| CD206 Antibody, anti-human, APC-Vio® 770 | 130-126-623 |
| Isotype Control Antibody, mouse IgG1, APC-Vio® 770 | 130-113-759 |
| REA Control Antibody (S), human IgG1, VioGreen, REAfinity™ | 130-113-444 |
| REA Control Antibody (S), human IgG1, APC, REAfinity™ | 130-113-434 |
| REA Control Antibody (S), human IgG1, PE-Vio 770, REAfinity™ | 130-113-440 |
| REA Control Antibody (S), human IgG1, PerCP-Vio , REAfinity™ | 130-113-441 |
| REA Control Antibody (S), human IgG1, Vio Bright B515 REAfinity™ | 130-113-445 |
| REA Control Antibody (S), human IgG1, PE, REAfinity™ | 130-113-438 |

**Table S2.** MAGPIX Luminex XMAp technonoly (MERCK).

| MAGPIX Luminex XMAp technonoly (MERCK) | Reference |
| --- | --- |
| Human RANTES | HCRTOMAG-60k |
| Human Matrix Metalloproteinase MMP9 | HMMP2MAG-55K |
| Human Adipokine PAI-1 | HADK1MAG-61K |
| Human Angiogenesis/Growth Factor | HAGP1MAG-12K |
| Human EGF :M-CSF-IL-10/MCP3/IL-13/IL-17A/IL-1a/IL-1b/IL-4/IL-6/IL-7/IL-8/IP-10/MCP-1/MIP1a/TNF-a/VEGF | HCYTOMAG-60K |
| Human VCAM-1/ ICAM-1 | HCVD2MAG-67K |
| Human TIMP-1/TIMP-2 | HTMP1MAG-54K |
| Human Placental Growth Factor | HCVD1MAG-67K |
| Human Adipokine | HADK2MAG-61K |
| Human Macrophage migration inhibitory factor (MIF) | HCCBP1MAG-58K |
| Human C Reactive Protein (CRP) | HCVD3MAG-67K |

- EMD Millipore provides three Luminex® instruments to acquire and analyze data using two detection methods:
- Luminex® 200™ and FLEXMAP 3D® analysers, which are flow cytometry-based instruments that incorporate key xMAP® detection components such as lasers, optics, advanced fluidics and high-speed digital signal processors.
- The Luminex® (MAGPIX®) system, a CCD-based instrument that incorporates xMAP® capture and detection components.

The ability to add multiple conjugate beads allows multiple results to be obtained from each sample. xMAP® technology thus allows multiplexing of many types of bioassays which reduces time, labour and cost compared to traditional assays.

Lysis of red blood cells

Analysis of the immune cell population by flow cytometry was performed at baseline, week 4 and week 12 after the start of irradiation.

Red Cell Lysis Buffer (RBC) is diluted from 10X to 1X working concentration with DI water. Pellets are then resuspended in 3 ml of 1X RBC Lysis Buffer and incubated on ice for 5 minutes.

Cell lysis is then stopped by adding 10 ml of Cell Staining Buffer to the tube. After 5 minutes in the centrifuge at 350xg the supernatant is discarded.

Viable cells are counted and resuspended in Cell Staining Buffer at 5-10 x 106 cells/ml. and 100 µl/tube of cell suspension (5-10 x 105 cells/tube) is dispensed into 12 x 75 mm plastic tubes.

Reagents that block Fc receptors can be used to reduce non-specific immunofluorescent staining. Cells must be pre-incubated with 0.25 µg of TruStain FcX™ PLUS (anti-mouse CD16/32) antibody per 10^6^ cells in a 100 µl volume for 5-10 minutes on ice.

Staining the cell surface with antibodies

Appropriately conjugated fluorescent, biotinylated or purified primary antibodies are added at predetermined optimal concentrations, incubated on ice for 15-20 minutes in the dark and washed twice with at least 2 ml of Cell Staining Buffer by centrifugation at 350xg for 5 minutes (Table 2 supplementary data)..

Cell pellets are resuspended in 0.5 ml of Cell Staining Buffer, incubated on ice for 3-5 minutes in the dark.

Perform MACS flow cytometry activated cell sorting.
